# Understanding differences between summer vs. school obesogenic behaviors of children: the structured days hypothesis

**DOI:** 10.1186/s12966-017-0555-2

**Published:** 2017-07-26

**Authors:** Keith Brazendale, Michael W. Beets, R. Glenn Weaver, Russell R. Pate, Gabrielle M. Turner-McGrievy, Andrew T. Kaczynski, Jessica L. Chandler, Amy Bohnert, Paul T. von Hippel

**Affiliations:** 1 Department of Exercise Science, Arnold School of Public Health, University of South Carolina, 921 Assembly Street, 1st Floor Suite, Room 131, Columbia, SC 29208 USA; 2 Department of Health Promotion, Education and Behavior, University of South Carolina, 915 Greene Street, Columbia, SC 29201 USA; 3 Medical University of South Carolina, College of Nursing, 99 Jonathan Lucas Street, Charleston, SC 29425 USA; 4 Department of Psychology, Loyola University Chicago, 1032 West Sheridan Road, Chicago, IL 60626 USA; 5 The University of Texas at Austin, Lyndon B. Johnson School of Public Affairs, 2300 Red River Street, Austin, TX 78712 USA

**Keywords:** Children, Obesity, School, Summer

## Abstract

**Background:**

Although the scientific community has acknowledged modest improvements can be made to weight status and obesogenic behaviors (i.e., physical activity, sedentary/screen time, diet, and sleep) during the school year, studies suggests improvements are erased as elementary-age children are released to summer vacation. Emerging evidence shows children return to school after summer vacation displaying accelerated weight gain compared to the weight gained occurring during the school year. Understanding how summer days differ from when children are in school is, therefore, essential.

**Discussion:**

There is limited evidence on the etiology of accelerated weight gain during summer, with few studies comparing obesogenic behaviors on the same children during school and summer. For many children, summer days may be analogous to weekend days throughout the school year. Weekend days are often limited in consistent and formal structure, and thus differ from school days where segmented, pre-planned, restrictive, and compulsory components exist that shape obesogenic behaviors. The authors hypothesize that obesogenic behaviors are beneficially regulated when children are exposed to a structured day (i.e., school weekday) compared to what commonly occurs during summer. This is referred to as the ‘**Structured Days Hypothesis’** (SDH). To illustrate how the SDH operates, this study examines empirical data that compares weekend day (less-structured) versus weekday (structured) obesogenic behaviors in U.S. elementary school-aged children. From 190 studies, 155 (~80%) demonstrate elementary-aged children’s obesogenic behaviors are more unfavorable during weekend days compared to weekdays.

**Conclusion:**

In light of the SDH, consistent evidence demonstrates the structured environment of weekdays may help to protect children by regulating obesogenic behaviors, most likely through compulsory physical activity opportunities, restricting caloric intake, reducing screen time occasions, and regulating sleep schedules. Summer is emerging as the critical period where childhood obesity prevention efforts need to be focused. The SDH can help researchers understand the drivers of obesogenic behaviors during summer and lead to innovative intervention development.

## Background

In the United States (U.S.), the prevalence of obesity among children aged 6–11 years has increased substantially in the last five decades [1], with the most recent estimates indicating 31.8% of children and adolescents aged 2–19 years are classified as either overweight or obese [2]. Children who are overweight or obese are at an increased risk for developing non-communicable diseases [3], thereby establishing childhood obesity as an immediate public health concern [4, 5]. Intervention strategies targeting obesity prevention among youth have focused primarily on four obesogenic behaviors: increasing physical activity (PA), decreasing sedentary/screen time, and improving dietary intake and sleep length and patterns. There is consistent evidence that these behaviors, alone or in combination, are associated with unwanted weight-gain in children [6–9].

The majority of the literature describing or intervening on ‘obesogenic behaviors’ of youth has been conducted during the 9-month school year, hereon referred to as ‘school’. The rationale for this is straightforward – over 90% of youth in the US attend public or private schools for approximately 6 h each day, 180 days of the year [5]. However, a number of recent studies consistently show that when children return to school after summer they display accelerated weight gains compared to the weight gain occurring during the school year [6, 10–15]. In addition, children display a loss in cardio-respiratory fitness (CRF) over the summer compared to the school year [16, 17]. These negative health outcomes are more pronounced in children who are already overweight or obese, of ethnic minority, and from low socio-economic-status (SES) households [11].

Investigations into the causal factors associated with the accelerated weight gain and loss in CRF during summer are limited and report mixed findings [13, 18, 19]. Studies report that children are more active [19], while others report they are less active during summer compared to school [13]. Studies report children have higher screen time during summer compared to school [18, 20], have less favorable diets or similar dietary intake during summer compared to school [13, 19], and sleep either the same amount [18] or slightly less [21] during summer compared to school. These studies were limited by study-design (e.g., between-subjects), definitions of “summer” (e.g., summer, winter, and holiday break data combined), obesogenic behavior assessment (e.g., self-report measures), and/or sample size (e.g., repeated measures on 14 children). These limitations prohibit the understanding of the causal factors associated with the occurrence of accelerated weight gain during summer.

Although convincing evidence documents the accelerated weight gain and loss of fitness during summer, there is currently a lack of frameworks or working hypotheses articulating the substantive differences between summer versus the school year that may lead to negative health outcomes. Conceptually, a fundamental difference in a child’s day during the school year versus summer is the presence of a consistent, structured, less autonomous (compared to summer), and segmented day with adult supervision. School days are an example of an ever-present structured environment with purposive, segmented, restrictive, and compulsory components. Conversely, summer days, for the most part, can be viewed as an environment with less formal structure and a higher degree of open-endedness. Subsequently, a more autonomous environment (e.g., summer) provides children with greater choice and the environment within which greater choice may exist – such as the home environment – has both physical and social aspects that can negatively influence a child’s weight status, particularly in children from low-income households [22, 23]. On the contrary, it must be noted that children are not without choice during more structured and regulated environments, like a school day; and research shows how children will select the less-healthful option (e.g., unhealthy snack), knowingly so [24], in light of a more health-enhancing option [25].

In absence of a extensive literature base to draw from that investigates summer and school differences, we propose that a day during the summer can be considered analogous to a weekend day during the school year. Although weekends are shorter in duration in comparison to summer, they possess similarities in that children are largely free from segmented, restrictive, and compulsory daily components (compared to what school demands) and allowed to make more autonomous choices in their behaviors. Thus, identifying children’s obesogenic behaviors during a less-structured day (weekend day) during the school year and comparing this in relation to a structured day (weekday) might shed light on what occurs over the summer. Further, it is plausible that, in comparison to school, a *more* autonomous and *unhealthier* home environment operates, and, thus, allows children to self-select and indulge in a variety of unhealthy behaviors of which, compounded over an uninterrupted 3-month period, results in adverse health outcomes (e.g., accelerated weight gain) that are not manifested during weekends during the 9-months of the school year due to the shorter duration of this time period.

In this debate article, the authors put forward the ‘**Structured Days Hypothesis’** (SDH) which is founded on the premise that a structured day (represented herein by a school day), defined as a pre-planned, segmented, and adult supervised compulsory environment, plays an overall protective role for children against obesogenic behaviors, and, ultimately, prevents the occurrence of negative health-outcomes, in this case excessive weight gain and loss in CRF. Equally, the absence of ‘structure’ to summer days could be one of the reasons children return to school, after summer break, with accelerated weight gain and decreases in CRF. Within the confines of the SDH, the authors present the scientific evidence on PA, sedentary/screen time, diet, and sleep in relation to the larger literature base that compares weekdays versus weekend days – two contexts considered structured and less-structured environments, respectively.

## The ‘structured days hypothesis’ (SDH)

**Fig. 1:**
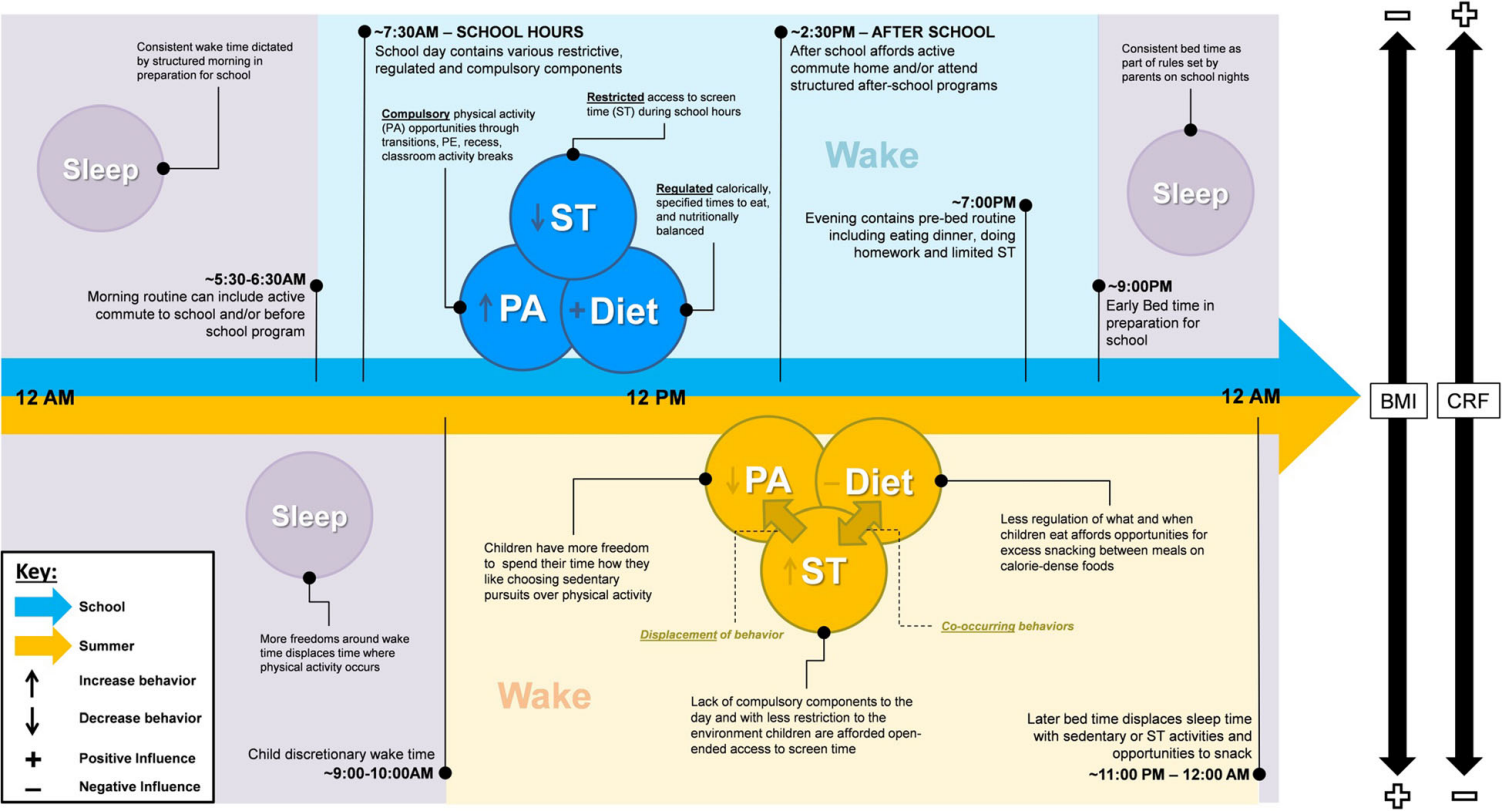
Structured day hypothesis conceptual model

In the SDH, it is hypothesized that the consistent presence of structure, routine, and/or regulation within a day positively shapes the obesogenic behaviors of youth. It must be noted that structure, regulation, and/or routine is neither a novel concept nor a new experience for children, as children are exposed to such traits on a day-to-day basis for the majority of the calendar year (i.e., a school day). Further, the SDH draws from concepts found in the ‘filled-time perspective’ which is based on the principal that time filled with favorable activities cannot be filled with unfavorable activities [26]. This applies to the SDH where structure, routine, compulsory, and/or regulation – common characteristics of a school day – fills children’s time with ‘favorable activities’ such as scheduled PA opportunities (e.g., school PE, recess) and regulated caloric intake (e.g., school meal programs) and set meal/snack times. Figure 1 illustrates a conceptual model of the SDH. During summer, the SDH proposes there is less structure, routine, and/or regulation, and more autonomy for children during summer afforded in the home environment. This leaves more time that can be filled with unfavorable activities/behaviors that are more prevalent in the home environment, such as extended periods of sedentary/screen time and/or liberties to choose when, what, and how much to eat/drink [22, 23, 27]. Importantly, during less-structured environments unfavorable activities can displace favorable activities (e.g., children choose sedentary pursuits over PA) and co-occur (e.g., snacking whilst watching TV). In addition, structured days (i.e., a school day) provide all children with opportunities and exposure to more favorable obesogenic behaviors (e.g., PA opportunities, restricted screen time), and a lower degree of child autonomy (e.g., limited eating occasions and choice of foods/beverages) compared to less-structured days (i.e., a summer day). Using the school day as an example of a structured environment, the proposed mechanisms for how the SDH operates across PA, sedentary/screen time, sleep, and diet, are detailed below. A summary is also provided in Table 1.

1. **Physical Activity:** The authors hypothesize school days (i.e., typical weekday) are fundamentally different from less-structured days, such as a weekend day or summer days, due to the fact that they consistently contain a daily structure and routine with intentional (e.g., recess, physical education, before/after school programs, organized sports programs) and unintentional (e.g., regular transitions between activities, walking to school) PA opportunities provide to the majority of children through the school day [28]. For example, a child may be exposed to all or a combination of some of the following on a school day; a morning commute to school, recess, physical education, lunch recess, after school program or activity, organized sport program, and a commute home from school [29]. Hypothetically, during less-structured days there may be less daily pre-planned PA opportunities for children, and the less-structured nature of the day itself reduces the occurrence of unintentional PA opportunities. Further, increased autonomy during less-structured days may allow children to choose to sedentary pursuits over physical activity (*displacement*).
2. **Sedentary/Screen Time:** The authors hypothesize the routine structure of a school day limits the amount of time children can spend sedentary, such as when watching TV or playing video games. Although children can spend a large amount of time sitting during the school day, bouts of time spent sedentary are broken-up by transitions during the segmented day and by planned opportunities where minimal sedentary time can occur (e.g., physical education, recess) [30]. Conversely, during less-structured days – where there may be less regulation or restriction –children may be exposed to increased unsupervised and open-ended periods of time where they are free to indulge in sedentary activities, such as TV viewing and playing computer games [11, 12]. In addition, co-occurring unfavorable behaviors are freer to operate, such as snacking whilst watching TV.
3. **Sleep**: The authors hypothesize the presence of the structured school day plays a role in minimizing the shift of bed/wake times. Specifically, children are going to bed earlier and waking earlier during school days, which studies have found is more beneficial to a child’s weight status than a late bedtime/late waketime [31]. For example, on a night that precedes a school day there is more likely to be a consistent bed-time and corresponding wake-time, followed by a typical morning routine (i.e., structure). This is incidentally enforced as a result of the presence of the school day. During a less-structured day, such as summer, there may be less structure in a child’s morning and evening periods; where children may be given more freedoms to stay up later in the evening and wake later in the morning. The later bed and wake times displace time that could be spent engaging in favorable obesogenic behaviors such as sleep in the evening, and PA in the morning, respectively.
4. **Diet:** The authors hypothesize children have limited opportunities to eat/drink during the school day [12] and access to regulated food programs [11, 15] that provide nutrient dense meals that meet existing federal nutrition guidelines [32]. Conversely, less-structured days (e.g., weekend day, summer day) may be giving children increased opportunities to snack and access to unhealthier foods in the home. As summer may present an open-ended and autonomous environment for children, other factors could drive increased energy intake during summer such as increased snacking of calorie-dense low nutrient foods whilst engaging in screen time activities for extended periods of time (*co-occurring behavior*) [33, 34].

**Table 1:** How Structured Days Hypothesis (SDH) operates across obesogenic behavior

Obesogenic Behavior	Protective Element of ‘Structured’ School Day	Impact on Obesogenic Behavior	School Example(s)
Physical Activity	Compulsory and voluntary PA opportunities for physical activity to occur before, during, and/or after school.	Increased daily PA	• Walking to/from school • Recess • Physical education • Transitions between class/activities • Classroom PA Promotion • Before/after-school programs or sports clubs
Sedentary/Screen Time	Segmented school day limits amount of time children spend seated. Limited non-educational screen time.	Decreased daily sedentary/screen time	• Bouts of sedentary time broken-up by transitions in and to/from class • Classroom teachers control screen time exposure
Sleep	Presence of school day establishes consistent early bed/wake times for children and evening/morning routines and rules.	Earlier bed/wake times	• Parent/Guardian enforces earlier bed/wake time rules on school days • Child is awake earlier to engage in morning intentional and unintentional PA • Early bed time reduces child engagement in sedentary/screen time
Diet	Structure of school day limits eating occasions for children. Schools offer regulated access to nutrient dense meals.	Decreased access to unhealthy foods/beverages and reduced overconsumption	• Scheduled opportunities to consume foods/beverages in school (e.g., breakfast, recess, lunch). • NSLP serve nutritionally balanced, age-appropriate portions.

*NSLP* National School Lunch Program

To support these hypotheses, a systematic search following the PRISMA guidelines [35] of published studies reporting week day and weekend day obesogenic behaviors of elementary-age children ages 5 to 11 years was conducted. A separate search, using PubMed, Google Scholar, and Web of Science, was performed for each of the following obesogenic behaviors: PA, sedentary, screen time, sleep, and diet. The following key words and/or search terms were used: “*physical activity*” sedentary*, screen*, television*, sleep*, diet*, nutrition*. Each of these individual terms were followed by weekday* weekend* and child*. Studies were excluded if they investigated a different population (e.g., adolescents, adults, children with disabilities), did not report weekend versus weekday outcomes, and/or were not published in English. Studies reporting outcome data of children that incorporated other ages outside 5–11 year old range were included as long as data were segmented by age group/category. Included studies were stratified by country (U.S. or International) and whether or not a statistical test was carried out on the difference between the weekday and weekend day outcome (statistical test or no test). Studies showing statistically significant (as defined within each study) favorable outcomes (e.g., increased PA, reductions in sedentary/screen time, earlier bed/wake times, and lower consumption/frequency of consumption of unhealthy foods/drinks) during weekdays (i.e., structured days) compared to weekend days (i.e., less-structured days) were classified as for the SDH. If a study reported outcomes that did not align with the above criteria (e.g., Weekend days more favorable than weekdays or no difference) was classified as against the SDH. Table 2 presents the number of studies found, excluded, and if the weekend day versus weekday outcome was for or against the SDH.

**Table 2:** Weekend Day (WE) vs Weekday (WD) evidence supporting the Structured Days Hypothesis (SDH) in elementary school-aged children

Obesogenic Behavior	Structured Days Hypothesis (SDH)	Number of Studies:											References
		SEARCH	EXCLUDED				USA STUDIES (INTERNATIONAL STUDIES)						
			Different population	WE vs. WD not reported	Foreign language	Total Excluded	TOTAL INCLUDED		FOR SDH		AGAINST SDH^b^		
							Statistical Test	No Test	Statistical Test	No Test	Statistical Test	No Test	
**Physical Activity**	Lower levels of PA during WE vs. WD	339	171	70	7	248	*15* (49)	*3* (24)	*13* (39)	*2* (20)	*2* (10)	*1* (4)	[29, 36–53, 55–120]
**Sedentary/Screen Time** ^**a**^	More sedentary/screen time during WE vs. WD	256	126	62	6	194	*6* (29)	*5* (22)	*6* (22)	*3* (16)	*0* (7)	*2* (6)	[30, 39, 46, 47, 63, 65, 67, 72, 73, 77, 79, 89, 93–95, 99–101, 103, 104, 106, 108, 111, 112, 115, 121–149]
**Sleep**	Later bed/wake time during WE vs. WD	133	73	31	7	111	*0* (0)	*6* (16)	*0* (0)	*6* (16)	*0* (0)	*0* (0)	[153–174]
**Diet**	Consume unhealthy foods/beverages more frequently during WE vs. WD	139	84	35	5	125	*8* (7)	*0* (0)	*9* (4)	*0* (0)	*0* (3)	*0* (0)	[34, 68, 113, 128, 175–185]
**Subtotal of Studies – USA** (International)							*29* (85)	*14* (62)	*27* (65)	*11* (52)	*2* (20)	*3* (10)	
**Total Number of Studies**		**867**	**454**	**198**	**25**	**677**	**190**		**155**		**35**		

^a^Combined results of two separate literature searches completed for sedentary* and screen*

^b^Reported statistical outcome either null or in opposite direction (*p*-value 0.05)

## Results

In total, 867 studies were screened for inclusion. Based on the exclusion criteria, 677 studies were removed because of sample population (*n* = 454), omission of a weekend vs weekday outcome (*n* = 198), or not published in English (*n* = 25). Of the remaining studies included for assessment (*n* = 190), there was no indication of patterns or groupings for studies that were categorized as either for or against the SDH in terms of study characteristics (e.g., method of obesogenic behavior measurement, sample size, country/continent of origin).

### Physical activity

A total of 91 studies reported weekend day and weekday PA estimates, with 18 originating from the U.S. [36–53]. Of these, 81% reported findings supporting the SDH. Two recent U.S. based studies employing objective measures of PA concluded that accumulated MVPA was higher on weekdays compared to weekend days. The first study explored 187 2nd and 3rd grade children’s (48.7% boys) MVPA on weekdays and weekend days using 5 days of accelerometer assessment. The authors reported that children’s MVPA was greater during weekdays (46.0 min/day) compared with weekend days (37.7 min/day) [36]. Another accelerometer-based study examined disparities in MVPA among overweight and obese 3rd – 5th grade children. Children classified as Overweight or Obese accumulated 11 min less of MVPA on weekend days compared with weekdays (*p* < 0.05) [37]. Similar trends have been found in the PA literature when using different objective measures of PA (e.g., pedometers [43, 44, 49]), self-report measures of PA [50], and investigating girls PA patterns [39, 54]. Further, a meta-analysis of objectively measured PA revealed school-aged children are more active on weekdays than weekend days (+14 MVPA min/day) [42].

Seventy three international studies were identified that report weekday and weekend day differences in PA [29, 55–120]. Of these studies, ~80% drew similar conclusions to the U.S literature, with 39 of these studies showing PA was lower on weekend days compared to weekdays reporting a statistically significant difference. One cohort study (*N* = 704) investigating seasonal variation in children’s PA was conducted in the United Kingdom (UK) and reported that across all seasons, accumulation of MVPA was higher on weekdays compared with weekend days [55]. The authors suggested that PA during weekday is less likely to vary as the school day – and its corresponding daily PA segments – are less likely to be influenced by seasonal changes, whereas weekend days are more susceptible to influence due to the volitional nature of PA opportunities. Another study measuring PA levels via accelerometry in a large sample of 11-year old children (*N* = 5595) found weekdays to be more active than weekend days (+31 cpm) [29], and other accelerometer-based studies conducted across different continents (e.g., Canada [56], Sweden [57], and Singapore [58]) in varying samples of elementary school-aged children (N ~ 80 to 1300) report MVPA is higher on weekdays compared to weekend days.

### Sedentary/screen time

A total of 62 studies were identified reporting either sedentary and/or screen time estimates for elementary school-aged children. Of these 62, ~18% of studies were conducted in the U.S. [30, 39, 46, 47, 121–127], with the remaining studies from other countries [63, 65, 67, 72, 73, 77, 79, 89, 93–95, 99–101, 103, 104, 106, 108, 111, 112, 115, 118, 128–149]. The majority of the sedentary/screen time estimates reported in the literature came from self-report measures (e.g., surveys, questionnaires, recalls). An early U.S. study analyzed TV viewing data from the Panel Study of Income Dynamics (PSID; 1997), collected using 24-h time diaries completed by the primary caregiver (i.e., parent/guardian). The analysis of ~1000 boys and girls (6–12 years) reported TV viewing increased on average by 60 min per day during weekend days compared to weekdays [121]. Another study reported that during weekdays 82% of children (*N* = 245; 6th to 8th grade) watched ≤2 h per day of TV (screen time recommendation for US children) compared to 76% of children on weekend days during school [122]. This finding is in agreement with several other studies conducted outside the U.S. [101, 134, 142, 150–152]. For example, Jago et al. (2014) examined survey data from parents of 5–6 year old children (*N* = 1078) on several screen time behaviors (e.g., TV viewing, computer use, videogame consoles). The percent of children spending ≥2 h per day engaged in screen time increased by approximately 34% during weekend days compared to weekdays [134]. Another study of approximately 15,000 children across multiple European countries reported 52% of the sample engaged in ≥2 h per day of screen time on weekend days compared to 20% on weekdays [140].

Twenty six studies reported the amount of time children spend sedentary comparing weekdays versus weekend days using objective measures (e.g., accelerometers, pedometers etc.). Atkin et al. (2016) analyzed seasonal data of 700 elementary school-aged children from a UK cohort study and reported increased sedentary time on weekend days compared to weekdays in 3 out of the 4 seasons (range; 9–54 additional sedentary minutes per day on weekend days) [55]. Another study explored in-school versus out-of-school sedentary time patterns of 206 5th grade children across 10 elementary schools in Colorado. The authors concluded children spent more time sedentary during weekend days (+5% of wear time) compared with weekdays [30]. In agreement, additional accelerometer-based studies from Canada [130, 132] and the United Kingdom [65, 108] concluded children spend statistically significantly more time sedentary on weekend days versus weekdays. Fifteen of the 62 studies found no difference in sedentary/screen time between weekend days and weekdays – or reported weekend days were less sedentary than weekdays.

### Sleep

A total of 22 studies reported bed and wake times for elementary school-aged children during weekdays and weekend days. Of these studies, ~27% were conducted within the U.S. [153–158], with the remaining 73% originating from other countries [159–174]. One of the earliest U.S. studies conducted by Blader and colleagues had parents of 978 5–12 year old children (85% Caucasian) complete a 48-item survey. The authors reported weekend bed/wake-times were 45–60 min later compared to weekdays [153]. A more recent study explored a large nationally representative sample of 3–18 year old U.S. children (*N* = 2281) and reported a clear shift in sleep time, with children going to bed and waking later on weekend days compared to weekdays across the range of ages [154]. Studies conducted outside the U.S. show similar bed/wake time patterns between weekdays and weekend days. Gulliford et al. (1990) conducted a cross-sectional study of British school children (*N* = 5145) whereby parents reported their child’s bed/wake-times [159]. The authors concluded children were going to bed and waking later on weekend days compared to weekdays starting at the age of 5 years old onwards. Several other international studies incorporating larger sample sizes (*N* > 15,000) and parent-report measures show similar findings [164, 165, 171], as do studies incorporating objective measures (e.g., accelerometers) to estimate bed/wake times [163, 170, 174].

### Diet

Fifteen studies reported elementary school-aged children’s dietary behaviors between weekend days and weekdays, with eight of these studies conducted in the U.S. [34, 175–181] Findings across U.S. based studies are consistent; children display statistically significant unfavorable diets on weekend days compared to weekdays. Baranowksi et al. (1997) reported children (*N* = 2984) had lower consumption of fruits and vegetables (FV) on weekend days compared to weekdays, with lunch time during weekdays identified as the eating occasion when children consumed the most FV [175]. This particular finding was supported by a more recent study that identified eating lunch from school was associated with higher overall diet quality compared with eating lunches from home [176]. Other studies have extended upon these findings and reported fewer FV were consumed on weekend days compared to weekdays, with children (*N* = 81; age range = 6-9 yrs.) consuming a greater percentage of calories from fat and non-nutrient dense snack foods on weekend days compared to weekdays [177]. This finding is consistent with an earlier study by Cullen and colleagues showing that, in comparison to weekdays, weekend days provided significantly more high-fat practices (e.g., choosing high-fat foods, adding fat to foods, preparing foods in fat), fewer low-fat practices (e.g., choosing lower fat foods, removing skin from chicken), and a higher percent of energy from fat when analyzing student-reported food records completed by 4th – 6th grade children (*N* = 520) from Texas [178]. Data from the National Health and Nutrition Examination Survey (NHANES 2003–2008) was analyzed to explore school meal participation in relation to dietary quality (*N* = 2376; 6–17 years old). Hanson et al. (2013) obtained dietary recalls and examined differences in Healthy Eating Index (HEI) scores for breakfast only and breakfast and lunch participants. Both categories of school meal participants (i.e., breakfast only and breakfast/lunch) had higher mean weekday HEI scores for milk and vegetables, and lower HEI scores for saturated fat and sodium compared to their HEI scores for weekend days [179]. Four out of seven international studies reported statistically significant findings with children displaying unhealthy dietary behaviors, such as increased sugar intake, during weekend days compared to weekdays [68, 113, 182, 183]. Three other international studies either found no difference [128, 184] or presented evidence showing favorable dietary behaviors during weekends compared to weekdays [185].

## Discussion

There is a clear need for further investigation in to children’s obesogenic behaviors during structured versus less-structured environments, none more so than school versus summer. The SDH presents the case that children require a structured environment to mitigate unhealthy behaviors from occurring. The evidence presented demonstrates that elementary school-aged children’s obesogenic behaviors are less favorable during less-structured (i.e., weekend days) versus structured days (i.e., weekdays). The findings herein support the argument that when elementary school-aged children are exposed to environments that contain less structure, regulation, and supervision, they indulge in a host of unfavorable behaviors. Typically, summer presents 3 months of the calendar year where a less-structured environment can exist for a prolonged period of time and the observed accelerated weight-gain and losses in CRF [6, 10–17] occurring during this window demonstrates the adverse impact a less-structured environment can have on children’s health and well-being.

Across all four obesogenic behaviors, 80% of the literature shows support towards the SDH. The structured nature of weekdays during the school year expose all children to various PA opportunities (e.g., recess, physical education, after-school programs, commute to school, classroom transitions/breaks) not necessarily guaranteed during weekend days. There is greater heterogeneity regarding the presence of structure and regulation on weekend days for children. This in turn may expose some children to environments where they are afforded greater autonomy over how they spend their time and/or present children with an environment that is constrained in opportunity to participate in favorable obesogenic behaviors, for example, children from low income households may not have access to PA programming and/or live in neighborhoods where crime is more prevalent, thereby limiting outdoor PA.

Nonetheless, the literature shows children’s obesogenic behaviors are beneficially-regulated during weekdays during the school year. This implies that intervention efforts should be focused on instances where a less-structured environment prevails, such as weekend days, winter breaks, and/or summer vacation. However, the authors would argue that weekend days during the school year may not merit intervention. Studies indicate that during the 9-month school year, increases in obesogenic behaviors during weekends and winter breaks do not impose the same detrimental effects on children’s health that summer does [6, 10–17], and this may be due, in part, to the short intermittent nature of weekend days in comparison to the prolonged duration of summer. The adverse weight and CRF outcomes associated with the presence of less-structured days (e.g., weekend days, winter breaks) are minimized or eliminated because they are interrupted by longer periods of exposure to a structured environment. For instance, over a typical 7 day week during the school year, only 2 of the 7 days are less-structured. Thus, less-structured environments, in and of themselves, may not be detrimental to weight gain and loss of CRF. Rather, we argue it is the duration of exposure to less-structured environments, as represented by summer vacation, which leads to accelerated weight gain and loss of CRF. In support, research has shown when children are exposed to a year-round structured environment interjected by short periodic breaks (e.g., year-round schools), they display a steady flat lining of BMI, particularly overweight and obese children [186]. Summer represents approximately a quarter of the calendar year, and this concentrated, largely non-interrupted exposure to a less-structured environment appears to be unfavorably impacting the health of children. This raises the question of whether summer is simply one long weekend?

In light of the SDH there are important implications to be considered by public health practitioners and researchers focused on tackling childhood overweight and obesity. A great deal of effort and resource has been allocated for intervening on and improving schools and other structured environments existing outside-of-school time (e.g., afterschool or sport programs) [5]. Reconsidering this strategy may be worthwhile given that structured environments, by the most part, appear to be doing a decent job of mitigating adverse health outcomes from occurring in children. As mentioned previously, in comparison to school, summer is a time where children have more autonomy and access to fill their time with unfavorable activities, particularly in the home environment. Even when autonomy is minimized, children inherently opt for the less-healthful alternative (e.g., unhealthy snack, sedentary activity) [25, 187, 188] and the home environment represents a more open-ended and less-regulated environment for children to overindulge in unhealthy behaviors that have been associated with overweight and obesity in children [22, 23, 189]. Thus, the potential for children to adversely impact their health is much greater during summer compared to when children are in a more structured and controlled environment (e.g., school). The authors wish to reiterate that the argument herein is not for the removal of free time or unstructured time for children to play. In fact, there are several examples of existing environments that are bound within structured days (e.g., recess at schools, free time at afterschool programs, sports clubs, and summer day camps) that offer children choice(s) on how to spend their time. The day-to-day schedule and segments of these programs will differ, regardless, it is the mere presence of these programs that is providing structure to a child’s day, and therefore, moderating the occurrence of unhealthy behaviors.

Given children likely spend more time at home during summer than during the school year, it is important to consider whether interventions targeting the home environment are the solution? Home-based childhood obesity interventions are limited in number and inconclusive in their effects [190] and can be a challenging and resource-consuming endeavor for practitioners [191]. Further, low-income and ethnic/racial minority households, a sub-population identified as having children most-at-risk for accelerated weight gain during summer [11], are susceptible to other economic and environmental factors (e.g., less income/access to purchase quality foods for family, safe neighborhoods for outdoor play etc. [192]) that may limit the success of home-based intervention strategies. An alternative and intuitive approach is to provide children with more opportunities and access to summer structured programs. When children spend summer days in a more-structured environment (e.g., summer day camp or program) they display favorable obesogenic behaviors compared to a less-structured environment [193, 194]. Public health practitioners and policy makers need to consider the benefit of structure to a child’s day and put more effort and resources into developing strategies and partnerships with community stakeholders to provide all children equal opportunities and access to summer structured programs. Overcoming pertinent barriers (e.g., cost) that isolate children from these structured settings is of paramount importance, with a recent American Camp Association report revealing approximately 75% of youth attending camps in the U.S. were from middle-to-high income households and Non-Hispanic White [195].

## Conclusions

In conclusion, the SDH posits that the school environment as a whole plays a protective role against the onset of unfavorable health outcomes by regulating obesogenic behaviors through its daily structure, regulation, and compulsory components. Within the last decade, researchers have identified summer as a time period where children are at risk of accelerated weight gain and losses in CRF [10–15], and the majority of this evidence stems from the U.S. Evidence showing negative health outcomes in children as a result of summer break in other countries has yet to be established, but one could speculate that a shorter summer break duration (e.g., United Kingdom, ~6 weeks) may not elicit the same detrimental impact on health outcomes in children as a longer summer break duration. Nonetheless, the authors argue that the SDH operates in a similar fashion, and the evidence from the international literature supports this with 117 out of 147 international studies showing obesogenic behaviors are more favorable on weekdays compared to weekend days. A key characteristic of both summer and weekend days is that, typically, both contexts have less consistent and formal daily structures, regulatory components, and present a more autonomous environment to children, unlike their counterparts (i.e., weekdays during the school year). However, the key element that distinguishes weekend days from summer days is the prolonged and concentrated period of time children are exposed to a less-structured environment. Summer is clearly the critical period where obesity prevention efforts need to be focused. The SDH provides a framework that can assist researchers and public health practitioners better understand the expression of obesogenic behaviors during less-structured environments, such as summer, and aid with the development of innovative observational studies and future intervention strategies.
